# Analysing the attributes of Comprehensive Cancer Centres and Cancer Centres across Europe to identify key hallmarks

**DOI:** 10.1002/1878-0261.12950

**Published:** 2021-03-30

**Authors:** Sebastian Kehrloesser, Simon Oberst, Willien Westerhuis, Astrid Wendler, Anke Wind, Harriët Blaauwgeers, Jean‐Benoit Burrion, Péter Nagy, Gunnar Saeter, Eva Gustafsson, Paolo De Paoli, József Lovey, Claudio Lombardo, Thierry Philip, Dominique de Valeriola, Marjet Docter, Femke Boomsma, Mahasti Saghatchian, Marek Svoboda, Irene Philip, Francesco Monetti, Henk Hummel, Gordon McVie, Renée Otter, Wim van Harten

**Affiliations:** ^1^ Cancer Research UK Cambridge Institute University of Cambridge Li Ka Shing Centre UK; ^2^ Organisation of European Cancer Institutes Brussels Belgium; ^3^ Cancer Research UK Cambridge Centre University of Cambridge UK; ^4^ the Netherlands Comprehensive Cancer Organisation (IKNL) Utrecht The Netherlands; ^5^ Rijnstate Hospital Arnhem The Netherlands; ^6^ Institut Jules Bordet Université Libre de Bruxelles Brussels Belgium; ^7^ National Institute of Oncology Budapest Hungary; ^8^ Division of Cancer Medicine Oslo University Hospital Oslo Norway; ^9^ Theme Cancer Karolinska University Hospital Stockholm Sweden; ^10^ Alliance Against Cancer Rome Italy; ^11^ Institut Curie Paris Cedex 05 France; ^12^ Radiotherapeutisch Instituut Friesland Leeuwarden The Netherlands; ^13^ Institut Gustave Roussy Villejuif France; ^14^ American Hospital of Paris Neuilly‐sur‐Seine France; ^15^ Masaryk Memorial Cancer Institute Brno Czech Republic; ^16^ Centre Léon Bérard Lyon France; ^17^ Ospedale Policlinico San Martino Genova Italy; ^18^ Cancer Intelligence Bristol UK; ^19^ The Netherlands Cancer Institute Amsterdam The Netherlands; ^20^ Department of Health Technology and Services Research University of Twente Enschede The Netherlands

**Keywords:** accreditation, clinical trials, comprehensive cancer center, multidisciplinarity, quality standard, translational research

## Abstract

There is a persistent variation in cancer outcomes among and within European countries suggesting (among other causes) inequalities in access to or delivery of high‐quality cancer care. European policy (EU Cancer Mission and Europe’s Beating Cancer Plan) is currently moving towards a mission‐oriented approach addressing these inequalities. In this study, we used the quantitative and qualitative data of the Organisation of European Cancer Institutes’ Accreditation and Designation Programme, relating to 40 large European cancer centres, to describe their current compliance with quality standards, to identify the hallmarks common to all centres and to show the distinctive features of Comprehensive Cancer Centres. All Comprehensive Cancer Centres and Cancer Centres accredited by the Organisation of European Cancer Institutes show good compliance with quality standards related to care, multidisciplinarity and patient centredness. However, Comprehensive Cancer Centres on average showed significantly better scores on indicators related to the volume, quality and integration of translational research, such as high‐impact publications, clinical trial activity (especially in phase I and phase IIa trials) and filing more patents as early indicators of innovation. However, irrespective of their size, centres show significant variability regarding effective governance when functioning as entities within larger hospitals.

## Graphical abstract

This study reveals the attributes of cancer centres based on data from 40 large European cancer centres, showing that Comprehensive Cancer Centres have significantly greater output of peer‐reviewed publications and clinical trials than other centres, and that the quality of multidisciplinarity is well established in all accredited cancer centres.

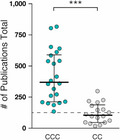

**Abbreviations**
A&D Programme: The Accreditation and Designation Programme of the Organisation of European Cancer Institutes
CC: Cancer Centre designation within the meaning of the Organisation of European Cancer Institutes’ Accreditation and Designation Programme
CCC: Comprehensive Cancer Centre designation within the meaning of the Organisation of European Cancer Institutes’ Accreditation and Designation Programme
EACS: The European Academy of Cancer Sciences
OECI: The Organisation of European Cancer Institutes

## 1 Introduction

Mortality pattern analyses across cancer registries in Europe and within individual member states have revealed significant differences in outcomes, which may indicate that patients may not have equal access to best practice, state‐of‐the‐art therapy or clinical trials [1].

In response to this challenge, we see in health systems across Europe a continuous process of concentration, cooperation in networks and formation of cancer centres^1^ within larger hospitals and university medical centres, strengthening clinical pathways and integrating with translational research and basic science. Recently, comprehensive cancer centres as hubs within wider ‘Comprehensive Cancer Infrastructures’ have been recommended to be an integral part of the EU Cancer Mission both by the Cancer Mission Board [2] and the European cancer community [3, 4, 5, 6, 7] to promote basic and translational research, to innovate in early detection and precision cancer medicine, and to meet the challenges of access to and quality of cancer care. More specifically, the Organisation of European Cancer Institutes (OECI) and the European Academy of Cancer Sciences (EACS) suggest that there should be one comprehensive cancer centre per 5–10 million people and at least one per member state, serving as quality drivers and network hubs within the national structures [3, 4, 5, 6, 7] and establishing a Europe‐wide network of cancer centres in order to close the quality gap [8, 9].

There are clear definitions of what defines a comprehensive cancer centre. Most fundamental is its multidisciplinary character and the governance as an identifiable entity, often within a larger structure. The comprehensive cancer centre definitions all involve the tripod of clinical care, cancer research and education. For instance, the Union for International Cancer Control (UICC) states in its 1978 document: *Guidelines for developing a Comprehensive Cancer Centre* that the scope of such a centre should encompass ‘clinical application of new knowledge for patient care, research which is both clinically and basically oriented, and […] professional and public education’ [10]. Aspects of cancer prevention are also a significant activity of most cancer centres in conjunction with public health, in that most of the robust oncogenetic services are to be found within cancer centres, as well as significant services directed towards the prevention of recurrence.

The characteristics of comprehensive cancer centres have been defined through accreditation programmes by OECI [11], the National Cancer Institute of the United States [12, 13] and Deutsche Krebshilfe [14], and these required characteristics show remarkable conformity. Within the cohort of Comprehensive Cancer Centres, EACS also defines the requirements of centres, which show the highest levels of research excellence [15].

There is growing evidence to suggest that aspects that characterise cancer centres such as high treatment volumes, earlier adoption of novel therapies, standardised treatment protocols and patient pathways, greater access to multidisciplinary consultation and easier access to clinical trials are in some domains related to superior outcomes for patients [16, 17, 18, 19, 20, 21, 22, 23, 24, 25]. Comprehensive Cancer Centres in particular are designed to bring together leading clinical expertise across all major cancer types with translational cancer research and education, thus generating mutual benefits, accelerating adoption of novel therapies and enrolment in clinical trials. However, the underlying evidence for the superior patient outcomes of Comprehensive Cancer Centres and other large cancer centres requires a greater evidential base [26].

In this study, we examine actual data from all 40 centres accredited between 2014 and 2020 by OECI. The 40 centres are located in 18 different European countries (15 out of 27 Member States of the EU plus Norway, Turkey and the UK) and include 22 centres that were subsequently designated as Comprehensive Cancer Centres^1^ (CCCs) and 18 designated as Cancer Centres^1^ (CCs) (Tables S1 and S2). They produce more than 12,400 peer‐reviewed publications on cancer research annually, have total annual research budgets of well over €1 billion and have treated more than 1 million new patients since their accreditations. We note that the OECI accreditation system is not the only such system in Europe: of note are also the Deutsche Krebshilfe accreditation programme for the 12–14 largest CCCs in Germany [14] and the Deutsche Krebsgesellschaft programme of organ centres and oncology centres throughout Germany and mainly neighbouring countries [27]. However, it should also be pointed out that the OECI A&D programme remains unique in the sense of assessing the comprehensiveness of cancer centres in a pan‐European manner.

The OECI Accreditation and Designation Programme is described in the Supporting Information. The OECI standards themselves have been accredited by the International Society for Quality in Healthcare, guaranteeing a rigorous process of objectivity and evaluation. As part of the latest revision of the quality standards, 100 European core quality standards for cancer care and research centres have recently been published [28].

The OECI Manual 2.0 [29, 30] is divided into six chapters:

Chapter 1: Leadership and management of the cancer centre
Chapter 2: Prevention and early diagnosis
Chapter 3: Cancer treatment and care
Chapter 4: Research, innovation and development
Chapter 5: Teaching and continuing education
Chapter 6: Patient centredness

The goal of this study was to identify the characteristics common to all cancer centres, and the particular features that distinguish CCCs. Although OECI centre designation status depends on predefined criteria (See Table S2 for these criteria) and data analysis may thus reinforce the specified categories, this analysis highlights common strengths and weaknesses in both categories, as well as divergencies, which can be used to derive learnings for emerging centres.

## 2 Data and Methodology

### 2.1 Data collection on 40 European cancer centres accredited by the OECI Accreditation and Designation Programme

The qualitative data we analysed relate to the auditors’ scoring of the degree of compliance with quality standards comprising 272 subquestions within the six chapters of the manual. The starting point for the analysis was the entire data set for the 40 centres, subsequently selecting for chapters, subchapters and individual standards where the scoring of the two cohorts (CCCs and CCs) showed statistically different results or wide ranges.

Quantitative data sets were selected (out of more than 800 possible metrics) based on their relevance to research, with the addition of some basic volume metrics.

We used the latest accreditation data set in every case. Data sets have been reviewed across all 40 centres to assure completeness and reliability. Outliers that were identified have been verified and manually curated by contacting the individual centres to validate the data. In view of the large number of items, we only report on significant findings in this paper.

### 2.2 Statistical analysis of qualitative and quantitative data

Individual (sub)‐standards in the qualitative questionnaires are scored by auditors as ‘yes’, ‘mostly’, ‘partially’ and ‘no’. Data are recorded as percentages of substandards (individual questions) answered with the particular score. In order to compare across centres, we calculated a compliance score using the following formula:

compliance score = ((2\% (yes) + \% (mostly) - \% (partially) - 2\% (no)) + 2Σ\% )/(4Σ\% )

This is a standard methodology where the goal is to arrive at a normalised score from 0 to 1, 1 being 100% of available answers being ‘yes’ and 0 being 100% of available answers being ‘no’. Normalisation to the sum of percentages answered no, partially, mostly or yes was necessary due to missing values or not‐applicable standards for certain centres. Data points with more than 33% of missing values have been excluded from the analysis for the particular (sub)chapter or standard.

Resulting compliance scores for chapters, subchapters and standards have been compared across CCCs and CCs using Welch’s t‐test to account for heteroscedasticity of the data and followed by the Bonferroni correction for multiple testing as indicated. Unbiased analysis was performed on chapters first, and if statistically significant differences between the two cohorts were found, analysis was then performed on subchapters, and thence to individual standards so as to identify the root sources of the differences between the centre types.

Quantitative data on 22 CCCs and 18 CCs have been compared using the Mann–Whitney test to account for non‐normal distribution of the data. Financial data in Euros were corrected for purchasing power parity (PPP) with EU28 = 1 [31].

## 3 Results

### 3.1 Study cohort characteristics – overall scale of OECI CCCs and CCs

**Fig. 1:**
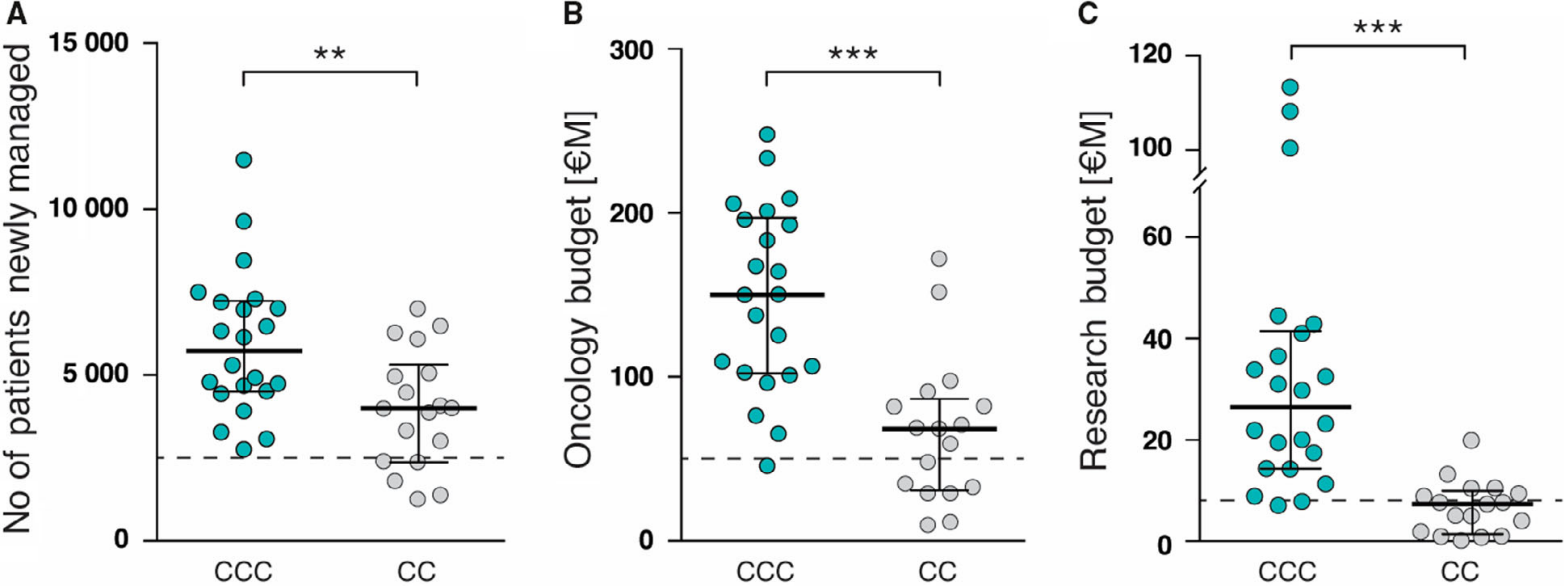
Size of OECI Comprehensive Cancer Centres (CCC) and OECI Cancer Centres (CC) in terms of patient numbers and financial budgets. (A) Number of patients newly managed (CCC *n* = 22, CC *n* = 18). All numbers reported for individual centres correspond to the index year of the accreditation process. (B) Oncology budget (CCC *n* = 22, CC *n* = 17) for cancer care. (C) Research budget (CCC *n* = 22, CC *n* = 17). Budget values have been converted to Euros and corrected for purchasing power parity. OECI thresholds for preliminary designation status for CCCs according to A&D Manual 2.0 are indicated with dashed lines. Middle horizontal lines represent the median, and bars represent interquartile ranges, ** P < 0.01, *** P < 0.001 (Mann–Whitney test).

CCCs are almost 50% larger in terms of newly managed patients (median 5,721) than the median CC (median 4,002) (Fig. 1A). The larger scale of oncology care in CCCs also translates to the median budget for oncology care (corrected for PPP) being more than twice as large in CCCs (median €150.1 M) compared with CCs (median €68.4 M) (Fig. 1B).

Consonant with the designation criteria of the OECI (Table S2), the difference between the two cohorts is more pronounced in the size of research budgets. CCCs have a median annual research budget of €26.5 M, whereas CCs have a median of €7.3 M (Fig. 1C).

### 3.2 Compliance with the OECI quality standards

**Fig. 2:**
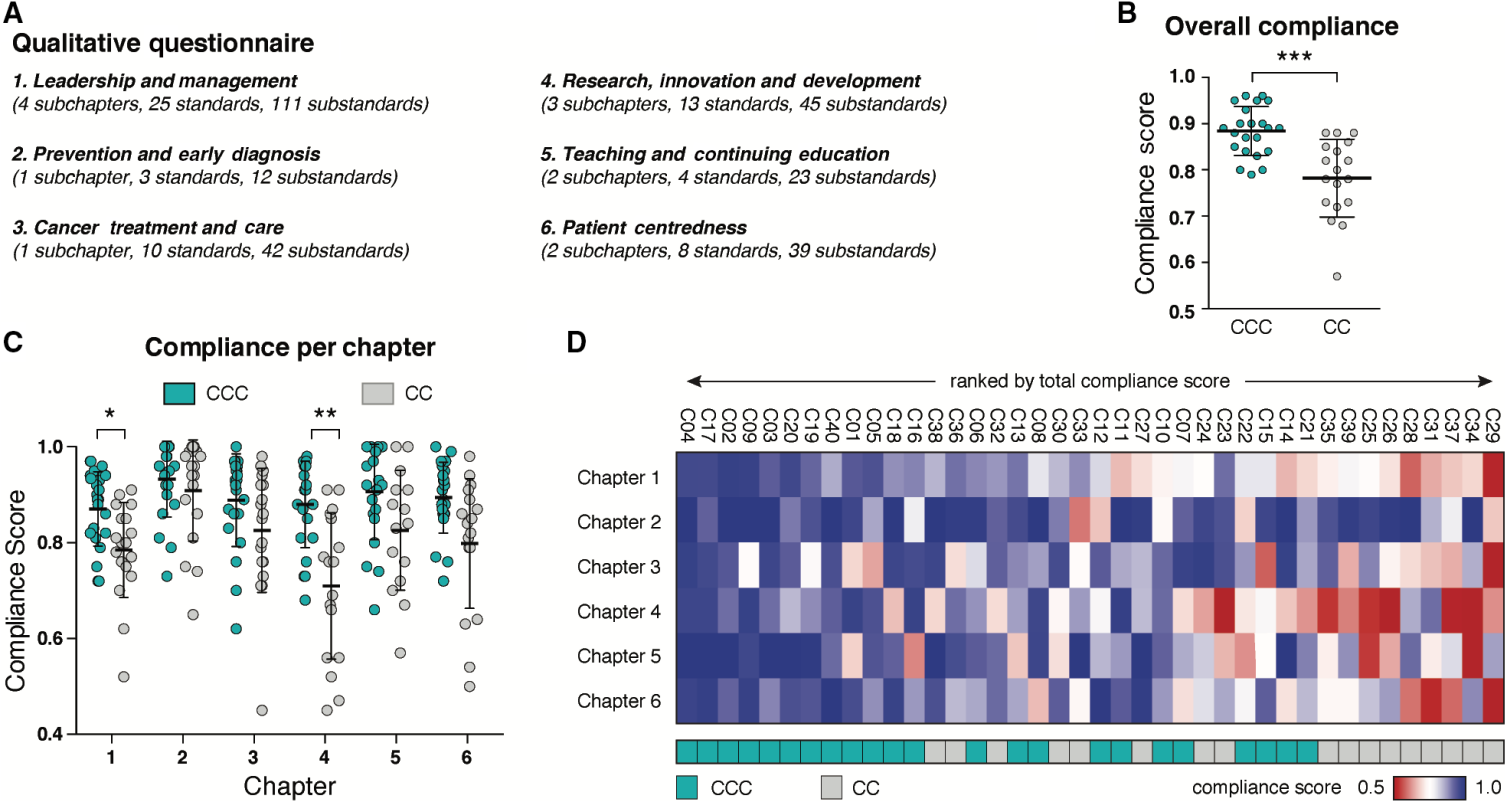
Centre compliance with OECI qualitative standards. Compliance has been compared between the two designation types across the six main chapters of the OECI qualitative questionnaire (A). Centres have been compared on overall compliance (B) and compliance with the individual chapters (C) (CCC *n* = 22, CC *n* = 18). A heat map of individual centre compliance and its designation status is shown in (D). Centres are ordered by rank of total compliance from left to right. Middle horizontal lines represent the mean, and error bars represent the standard deviation, * P < 0.05, ** P < 0.01 (Welch’s *t*‐test using the Bonferroni adjusted *P*‐values for multiple comparisons).

The overall compliance of both cohorts to the six chapters of qualitative items (Fig. 2A) showed that CCCs scored significantly higher overall (Fig. 2B). However, significant differences were only observed for chapters 1 (leadership and management) and 4 (research, innovation and development), with chapter 4 showing the largest absolute difference (Fig. 2C).

The corollary to this is that we find no statistically significant differences between the cohort of CCCs and that of CCs in the following domains: cancer prevention measures (chapter 2); cancer treatment and care standards (chapter 3); teaching and continuing education (chapter 5); and patient centredness (chapter 6).

CCCs are seen to consistently show high compliance across all categories, which is confirmed by the data when analysing compliance for individual centres (Fig. 2D).

In order to understand whether the observed difference between CCCs and CCs is due to specific standards within chapters 1 and 4, we compared subchapters and individual standards for all centres:

#### 3.2.1 Compliance with quality standards on leadership and management

Both CCCs and CCs show excellent compliance with operative aspects of chemotherapy (subchapter 1.2) and quality control processes for patient care (subchapter 1.4). However, both CCCs and CCs demonstrated slightly lower (but still good) compliance with standards on organisational structure and governance (subchapter 1.1), and on some operational aspects of care (subchapter 1.3) (Fig. S1A).

The largest absolute difference between CCCs and CCs and highest intracohort variability was observed for organisational structure and governance. Drilling down to individual standard level to analyse this (Fig. S1B and S1C), we see high variability for standard 1 (where we observe weaknesses in some centres’ compliance with questions on corporate strategic planning), for standard 5 (weaknesses in some centres regarding the quality of patient outcome data, and diagnostic trends reporting by centres, especially CCs) and for standard 6 (centres often lacked compliance with subquestions about management reporting and evaluating the effect of improvement actions).

Subchapter 1.3 contains standards concerning patient pathways and multidisciplinarity (Fig. S1D). Standard 17 assesses the overall documentation of patient pathways, where a complete documentation of patient pathways was not found in quite a few centres (especially CCs). More significantly, Standard 18 assesses the process of multidisciplinary team (MDT) meetings, where compliance data showed that the fundamental and practical aspects of MDT meeting processes and decision‐making are well established across almost all centres.

#### 3.2.2 Compliance with quality standards on research, innovation and development

Analysis of individual subchapters on research, innovation and development revealed strongly significant differences between CCCs and CCs in subchapters 4.2 (resources and materials) and 4.3 (process control) (Fig. S2A). We also observed considerable variation within the CCC cohort across all three subchapters.

To understand these differences, the next level of analysis was performed (individual standard level). Here, we found that CCCs were significantly stronger than CCs in research collaborations, organisation of clinical research, processes of intellectual property and innovation, and infrastructure for biobanking (Fig. S2B and S2C). CCCs were also more consistent in having a robust scientific knowledge transfer programme, being subject to regular external review, and in engaging an international Scientific Advisory Board. (Fig. S2D). We also observed that in these research standards, several CCs, and occasionally CCCs, showed low compliance with the standards, suggesting that processes and procedures around research required improvement.

### 3.3 Quantitative findings on research and innovation

**Fig. 3:**
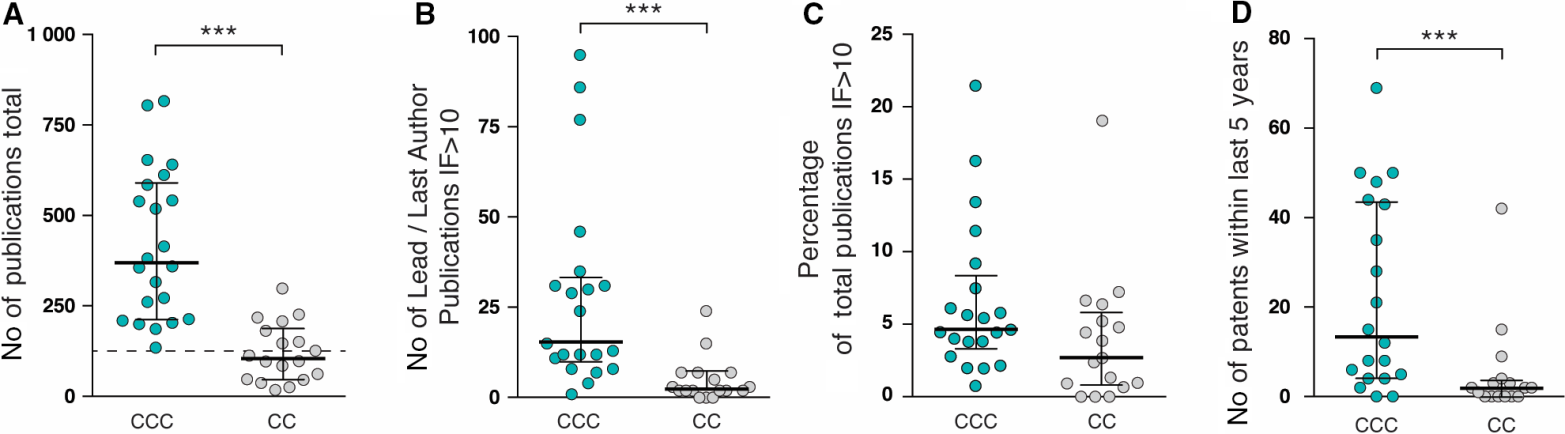
Research output in form of scientific publications and successful patent applications as early indicators of innovation. (A) Sum of all national and international publications in the index year (CCC *n* = 22, CC *n* = 18). OECI threshold of 125 publications for preliminary designation status for CCCs according to A&D Manual 2.0 is indicated with a dashed line. (B) Number of high‐impact publications (IF > 10) with the first or last author being a centre member in the index year (CCC *n* = 21, CC *n* = 17). (C) Number of high‐impact publications shown in (B) as a percentage of the total publication output shown in (A). (D) Number of filed or granted patents within the last 5 years. Middle horizontal lines represent the median, and bars represent interquartile ranges, ** P < 0.01, *** P < 0.001 (Mann–Whitney test).

**Fig. 4:**
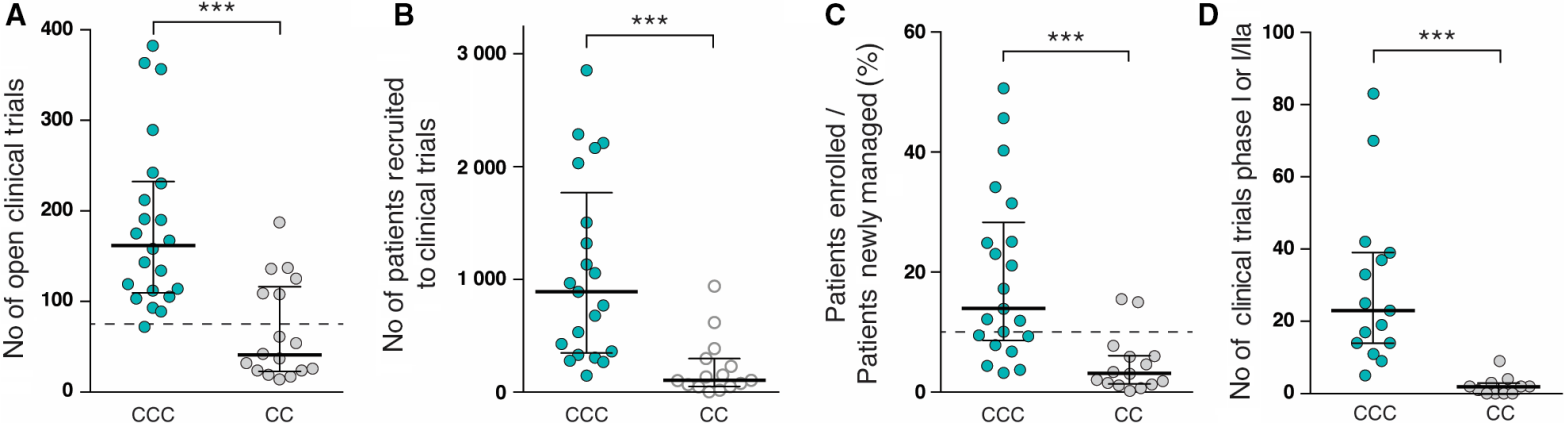
Clinical trial activity in OECI centres. (A) Total number of open clinical trials (interventional studies) (CCC *n* = 22 CC *n* = 17). (B) Total number of patients recruited to prospective interventional clinical trials within the index year (CCC *n* = 21, CC *n* = 15). (C) Patients recruited as a percentage of all newly managed patients (CCC *n* = 21, CC *n* = 15). (D) The number of open phase I and phase I/IIa trials is used as an indicator of centre initiated early clinical development (CCC *n* = 15, CC *n* = 11, data not available for A&D Manual v1.0). Dashed lines indicate OECI designation thresholds for CCCs: 75 open trials (A) and 10% of newly managed patients enrolled in prospective interventional trials (C). Middle horizontal lines represent the median, and bars represent the interquartile ranges, *** P < 0.001 (Mann–Whitney test)

The scale and quality of research, and its translation into clinical practice, was further measured by research outcomes in terms of scientific publications and patent applications filed or granted (Fig. 3) as well as clinical trial activity (Fig. 4).

#### 3.3.1 Scientific publications, and patents as early indicators of innovation

Higher research activity in CCCs results in a median total publication output of 370 national and international peer‐reviewed publications per year, compared with 104 for CCs (Fig. 3A), nearly a fourfold difference. Looking at the number of high‐impact publications (impact factor > 10) with first or last authorship in the centre, we see a more skewed distribution, with a few CCCs publishing significantly more high‐impact research compared with their peers (Fig. 3B). The absolute publication numbers show a good correlation with the annual research budget for the more research‐focused CCCs (Fig. S3). Although CCs have significantly fewer high‐impact publications, the percentage of high‐impact publications of the overall output is not significantly lower in CCs compared with CCCs (Fig. 3C).

The number of patents filed or granted within the last five years reveals that only a few centres appear to effectively support patenting or focus on patentable research (Fig. 3D) and these are mainly CCCs; we found a range of just a few to above 50 patents filed or granted.

#### 3.3.2 Clinical research activity

We find a very significant difference in the number of clinical trials open to recruitment in CCCs (median 162) compared with CCs (median 42) (Fig. 4A). However, a few CCs have more than 100 open clinical trials active. While the higher numbers of active trials also translate to higher patient numbers being recruited to prospective interventional trials per index year (median CCC 894, compared with median CC 123) (Fig. 4B), these numbers show a much greater variability (the upper interquartile is 1,144 for CCCs and 205 for CCs). When expressed as a percentage of newly managed patients in each centre per index year, we observe more consistent data, with the median CCC achieving interventional trial participation of almost 13.9% of its newly managed patients (Fig. 4C). This compares with a median of 3.1% of the CC cohort. However, not all CCCs consistently achieve the OECI designation threshold for CCCs of 10%, reflecting the fact that OECI looks at designation criteria ‘in the round’ (Table S2).

As an indication of the translational research activity within centres, the number of early phase I and phase I/IIa studies was counted (Fig. 4D). This reveals a significant difference between CCCs and CCs, while also showing striking variation among CCCs, ranging from only 5 to as many as 83 trials.

## 4 Discussion

The OECI A&D Programme’s data on 40 accredited large cancer centres enable us to identify common themes throughout all centres, and to identify some hallmarks/differentiators of CCCs and their particular contribution in cancer care, research and education:

### 4.1 Themes common to both cohorts (no statistically significant differences)

#### 4.1.1 Multidisciplinarity, pathway‐based and patient‐centred care

Our findings indicate that all cancer centres performed well in these domains, though CCCs generally scored more strongly and consistently. In particular, the functioning of MDT meetings in agreeing diagnosis and treatment recommendations, which was one of the innovations of cancer services in earlier decades, is now firmly established as a norm in all centres, and the representation of many professional disciplines at the MDT meetings is followed. However, a clear documentation of patient pathways is sometimes lacking, and we encountered different degrees of detail. Molecular Tumour Boards are now becoming a standard feature in cancer centres, but their existence was not consistently tracked in the OECI Manuals being reviewed here, although this measure is a clear feature of the new Manual 3.0. These, and other features of integrating latest scientific insights into patient care, are also evaluated by the European Academy of Cancer Sciences’ certification of Comprehensive Cancer Centres of Research Excellence [15].

Patient centredness at an individual patient level, offering choice, good information and clear communication, is common in most centres. The culture of involving patient groups in co‐creating practice developments is also becoming more firmly established. However, consistent analysis of patient outcomes and treatment trends was not adequate in many centres. OECI plans further research to analyse in greater detail the compliance with care‐related standards and patient centredness, and the implications of those findings for the future of cancer care in Europe.

#### 4.1.2 Challenges with centre governance and management

Our data showed that there is considerable variability in the quality of centre governance and management. Noncompliance with certain standards indicates that a common problem for centres is establishing an authoritative Cancer Board for the centre, which is adequately balanced between research and clinical care. This is much easier for standalone cancer centres; for centres within a University Hospital setting, needing to leverage the research of several institutes and University departments, this is a much greater challenge, which even some large CCCs appear to struggle with. In such settings, clinical and research accountability actually rests at a higher level than the Cancer Board. These challenges may also account for the apparent weaknesses in some centres in strategic planning and resourcing, which should clearly integrate research endeavours and clinical priorities. Research on how certain CCCs have been formed in recent decades within University Hospitals is being performed by OECI, which should help inform the future growth of key centres more evenly across Europe, especially in the context of the EU Cancer Mission.

#### 4.1.3 Comprehensive education programme

We have found that most centres score satisfactorily in this domain, though the analysis of compliance showed that there are underperformers in both cohorts. Evaluating the quality and breadth of educational programmes is notoriously tricky, and further analysis and incorporation of quantitative parameters would be necessary to measure and monitor the improvements in individual centres over time, and identify key areas for improvement.

### 4.2 Themes differentiating CCCs (statistically significant differences)

#### 4.2.1 Comprehensive research programme

Our findings show the degree to which CCCs leveraged breadth and depth in research. The median peer‐reviewed output of these institutions is nearly four times higher than its CC counterpart. CCCs also provide significant leadership in the authorship of high‐impact papers, the median being more than five times higher than that of the CC cohort, and this provides a substantial engine room for the understanding of cancer and the mechanisms of cancer therapy. This output is commensurate with the finding that the median research budget of CCCs is nearly four times higher than the median research budget of CCs.

#### 4.2.2 Integration of translational research into care

On clinical research, our data showed that the median CCC had nearly four times more prospective interventional clinical trials open to recruitment than its CC counterpart. As significantly, the rate of patient accrual to those trials is more than seven times the median of CCs. It would be important to understand the reasons for the difference between CCCs and CCs and how to improve on trial recruitment rates, which contribute to improved outcomes [32]. This may be an indication that only CCCs can adequately finance and support investigator‐led studies, and also an indication that pharma tends to concentrate their collaborations with CCCs for reasons of capability, efficiency or effectiveness. OECI does collect data on commercial trial activity compared with investigator‐initiated studies, but these data are not yet consistently curated. However, some very high recruiting rates indicate that the basis for reporting these numbers may not always have reflected only interventional studies (i.e. some centres may have mistakenly included some observational studies). Overall, the data show that when restricted to interventional therapeutic trials only, the 10% average recruitment rate is quite a high bar, even for some CCCs (the median rate was 13.9%). The most obvious disparity between the two cohorts concerned the number of early phase I and phase I/IIa studies open, where the median CCC managed 24 studies (with some striking variations within the cohort, from 5 to 83), compared with a median of 3 among CCs, some of which had none.

#### 4.2.3 Compliance with the qualitative criteria

Within the cohort of CCs, some examples of low compliance should be noted in Figs 2B, 2C, S1 and S2. These low scores relate in the main to the low volume of cancer research in these centres; in one CC case, the applicable legislation actually precluded research being conducted in that institution. For the reasons stated elsewhere in this paper, the clinical quality of these centres overall justified their accreditation as a CC, after appropriate improvement actions were implemented.

#### 4.2.4 Infrastructures linking research and the clinic

The quality of the infrastructure of biobanks, phase 1 trial units and biostatistical support for translational research is significantly superior in CCCs than in their CC counterparts, and these infrastructures should be regarded as vital building blocks of a comprehensive approach to cancer.

#### 4.2.5 Innovation, development and commercialisation strategy

Our findings indicate that CCCs were generally better resourced with technology transfer offices, support for intellectual property, commercialisation and had a greater focus on patentable research and patents that were filed or granted.

#### 4.2.6 Volume of clinical care

Our data show that while there are some large CCs in terms of newly managed patients per year, the median of the CCC cohort is almost 50% larger than its CC counterpart. But of greater interest for future research are the data showing that the oncology care budgets of CCCs show a much more pronounced spread at the top end, indicating the greater costs associated with large academic centres. Future research could examine the source of such greater costs, which seem to override economies of scale.

The strengths of this study include the extensive database that has been built up through the OECI Accreditation and Designation Programme. To our knowledge, no earlier studies involving so many different countries in Europe (18 countries) have been published, especially those using data from both care and research domains. A limitation is the possibility of differing interpretations of definitions and scores as a consequence of health system characteristics. Further improvements to data collection and curation would increase the value of the comparative analyses (for instance, clinical trial data).

It should be noted that this analysis of 40 accredited centres by OECI represents the total of accreditations in the period 2014–2020, and no centres have been excluded. During this period, accreditation has not been denied to any centre, which actually is accepted onto the programme; however, OECI has employed two policies: the first is to prescreen centres (including in some cases a previsit) on the basis of their basic oncology functions, governance and size. Those not meeting the prescreen criteria do not enter the programme and consequently do not incur abortive costs. The second policy, for those centres progressing in the programme, is to delay accreditation for a period of time after the peer review until key compliances are met – this was the case with 7 of the 40 centres reviewed in this paper. This policy is believed to be commensurate with idea of using an accreditation programme as a major tool to improve quality in a sustainable way. The peer‐review compliance scores in the figures reflect the evaluation of the audit teams immediately following the on‐site visit. In a number of cases, the low scores were addressed as key improvements, which had to be delivered by the centre prior to the accreditation decision. Explanations of some low compliance scores with chapter 4 (research) are given in 4.1 above.

The question of the geographical distribution of the CCCs and CCs accredited (listed in Table S1) is not the major focus of this paper, but is worthy of some note. The OECI accreditation scheme is a voluntary scheme relying on the motivation of an individual centre, except in the case of Italy, where, beginning in 2012, the Ministry of Health directed all major cancer research institutes to take part in the programme [33]. It should also be noted that Germany has a very complete accreditation coverage through the Deutsche Krebsgesellschaft programme [27] and the Deutsche Krebshilfe programme [14]. However, it is evident that in some eastern and southern EU member states the coverage of accredited CCs and CCCs is limited or nonexistent. This apparent gap is a major focus behind the proposals for Comprehensive Cancer Infrastructures recommended by the EU Cancer Mission Board [2]. It is evident that centres in some member states require expert advice and support at a local level, as well as a twinning function with other large accredited centres, to enable them to develop into high‐performing centres.

As noted in the Introduction, despite some seminal studies in the United States [17, 18, 24, 25], the superiority of treatment of equivalent patient cohorts in CCCs or large cancer centres over those in general hospitals has not yet been fully established. This is related to the obvious methodological challenges in outcomes research, especially the multifactorial contributors to better outcomes when comparing centres. Establishing a completely level playing field of equivalent cohorts based on cancer, stage of diagnosis, co‐morbidities, and social and economic indicators, in order to identify the specific contributions of the treating centre, is a major challenge when averaged across all cancers. So far, comparative studies have been successful only in the field of specific cancers, especially in relation to surgery and particularly related to volume differences [16, 19, 20, 21, 22, 23]. In the future, wider outcome studies related to the availability of molecular diagnostics and targeted treatments, and the impact of specialised and research‐active multidisciplinary teams in CCCs, would be welcomed.

There is then the wider question of how cancer centres disseminate their expertise to all settings diagnosing and treating cancer in a locality, thus addressing inequalities within countries. This challenge is addressed in outline in both the EU Cancer Mission [2] and Europe’s Beating Cancer Plan [9] in terms of developing and strengthening cancer networks and wider infrastructures. The outline of this challenge of Comprehensive Cancer Care Networks was set out in chapter 5 of the European Guide on Quality Improvement in Comprehensive Cancer Control [8]. OECI is addressing this challenge by piloting a set of quality standards for cancer networks, whose results will be published shortly.

## 5 Conclusion

With the EU Cancer Mission [2] and Europe’s Beating Cancer Plan [9] in the state of development at the date of writing, it is timely to review and recognise the hallmarks of cancer centres in Europe, and the particular characteristics of Comprehensive Cancer Centres. In particular, the Cancer Mission Board interim recommendation to establish ‘Comprehensive Cancer Infrastructures’ in every Member State of the EU will require a gap analysis of the current cancer infrastructures in Member States, and a plan to be agreed by Member States of how to develop more accredited cancer centres where these do not exist, and to build cancer networks/infrastructures both within and between Member States. This is supplemented by the flagship initiative in the Beating Cancer Plan to create by 2025 an EU network of Comprehensive Cancer Centres with a wide accessibility to EU citizens. The current study provides the first review of accreditation data on 40 of the largest European cancer centres. The analysis provides evidence that most OECI‐accredited centres have good compliance with care‐related quality standards, multidisciplinary working, professional education and patient centredness.

The main difference between CCs and CCCs lies in their comprehensive research programmes and clinical trial activity, and for the first time, we show the benchmarked evidence for this. The characteristics of CCCs are that they achieve higher output across the continuum of translational research, as assessed through the number of peer‐reviewed publications (especially those of high impact), clinical trial activity and number of filed or granted patents. On average, CCCs have established superior research infrastructure and achieve better integration of research and clinical care.

Surprisingly, the data reveal strong variation in compliance with leadership‐ and management‐related quality standards, even within the CCC cohort, and this variation justifies further research into what constitutes effective governance of cancer centres, as the EU Cancer Mission and Beating Cancer Plan encourages the creation and accreditation of Comprehensive Cancer Infrastructures in Europe.

## Conflict of interest

SK, SO and AW received funding from the Organisation of European Cancer Institutes for this work. WvH receives a nonrestricted grant from Novartis, a nonrestricted grant from Agendia BV and a nonrestricted grant from Intuitive Surgical. PN and JL acknowledge financial support from the Hungarian Thematic Excellence Programme (TKP2020‐NKA‐26). All remaining authors have declared no conflicts of interest.

## Author contributions

SK and SO jointly drafted this paper and analysis. WW, AW and HB were responsible for data collection and curation. WvH guided the paper strategically. J‐BB, PN, GS, EG, PDP, JL, TP, DdV, RO, AW and WvH contributed to the draft. All authors contributed to the OECI Programme on which this article is based.

## Dedication

The authors dedicate this paper with gratitude to the memory of our co‐author, Professor Gordon McVie (1945–2021): physician, scholar, innovator, campaigner, mentor and friend.

## Patient and Public Involvement

Patient and public involvement is a fundamental element of the OECI standards analysed by the paper, as disclosed in the results and discussion.

## Supporting information


**Fig S1.** Centre compliance to subcategories in chapters 1 – Leadership and Management.

**Fig S2.** Centre compliance to subcategories in chapters 4 – Research, Innovation and Development.

**Fig S3.** Correlation of publication output with research budget.

**Appendix S1.** Background to the Accreditation and Designation (A&D) Programme of the Organisation of European Cancer Institutes.
**Table S1.** List of accredited OECI Centres.
**Table S2.** List of accredited OECI Centres.

## Footnotes

^1^ In this paper, ‘cancer centres’ refers to all centres with a specialism in cancer, and ‘comprehensive cancer centres’ as generally understood in the literature, irrespective of OECI designation, while the capitalised terms Cancer Centre (CC) and Comprehensive Cancer Centre (CCC) refer to the OECI designation status
