# Supplementary figures and images for: Analysing the attributes of Comprehensive Cancer Centres and Cancer Centres across Europe to identify key hallmarks
Source: Mol Oncol. 2021 Mar 30;15(5):1277–88. doi: 10.1002/1878-0261.12950 (PMC8096787; doi:10.1002/1878-0261.12950)

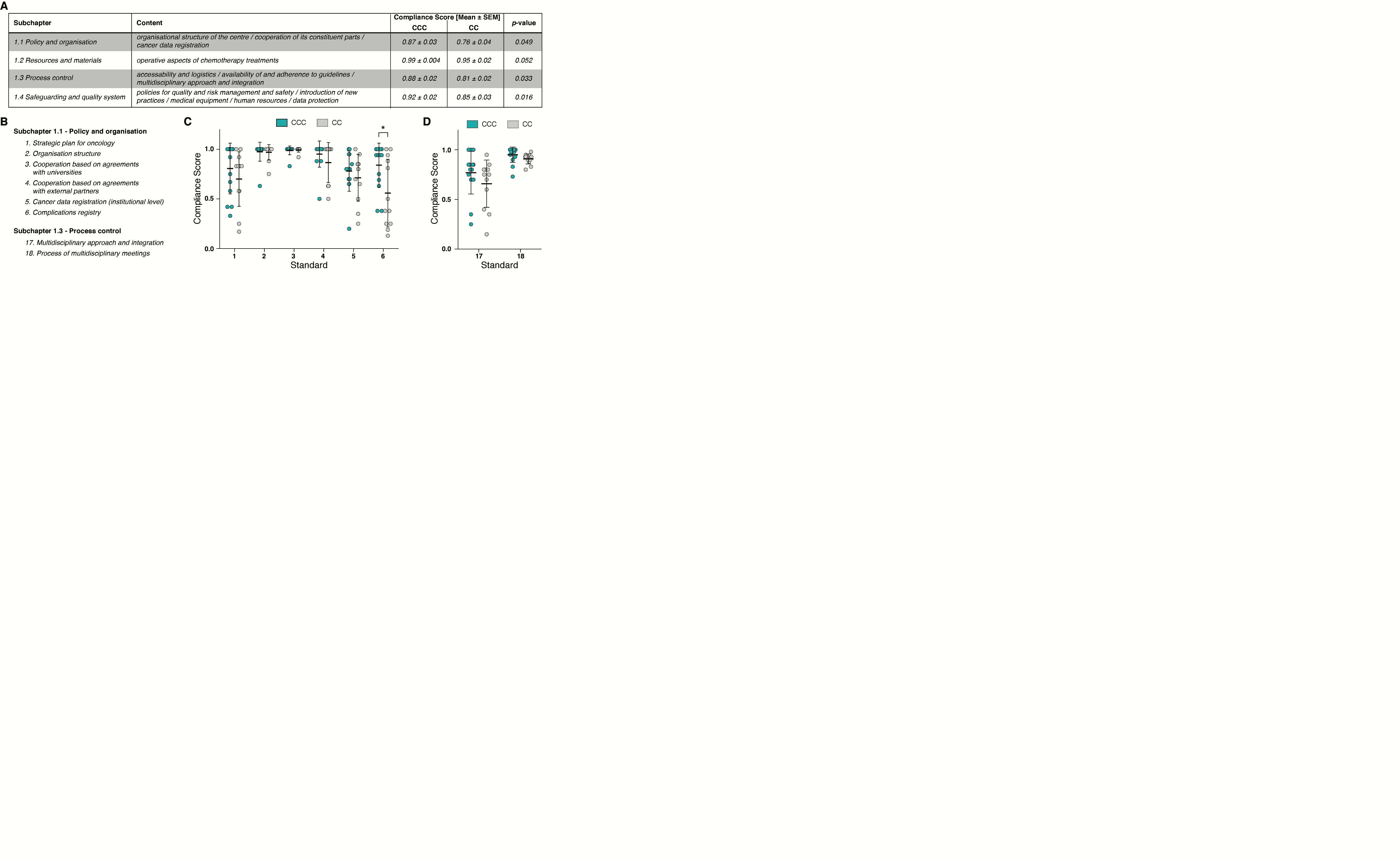

Supplement: Supplementary file 1 — Fig S1. Centre compliance to subcategories in chapters 1 – Leadership and Management. [file MOL2-15-1277-s001.tiff]

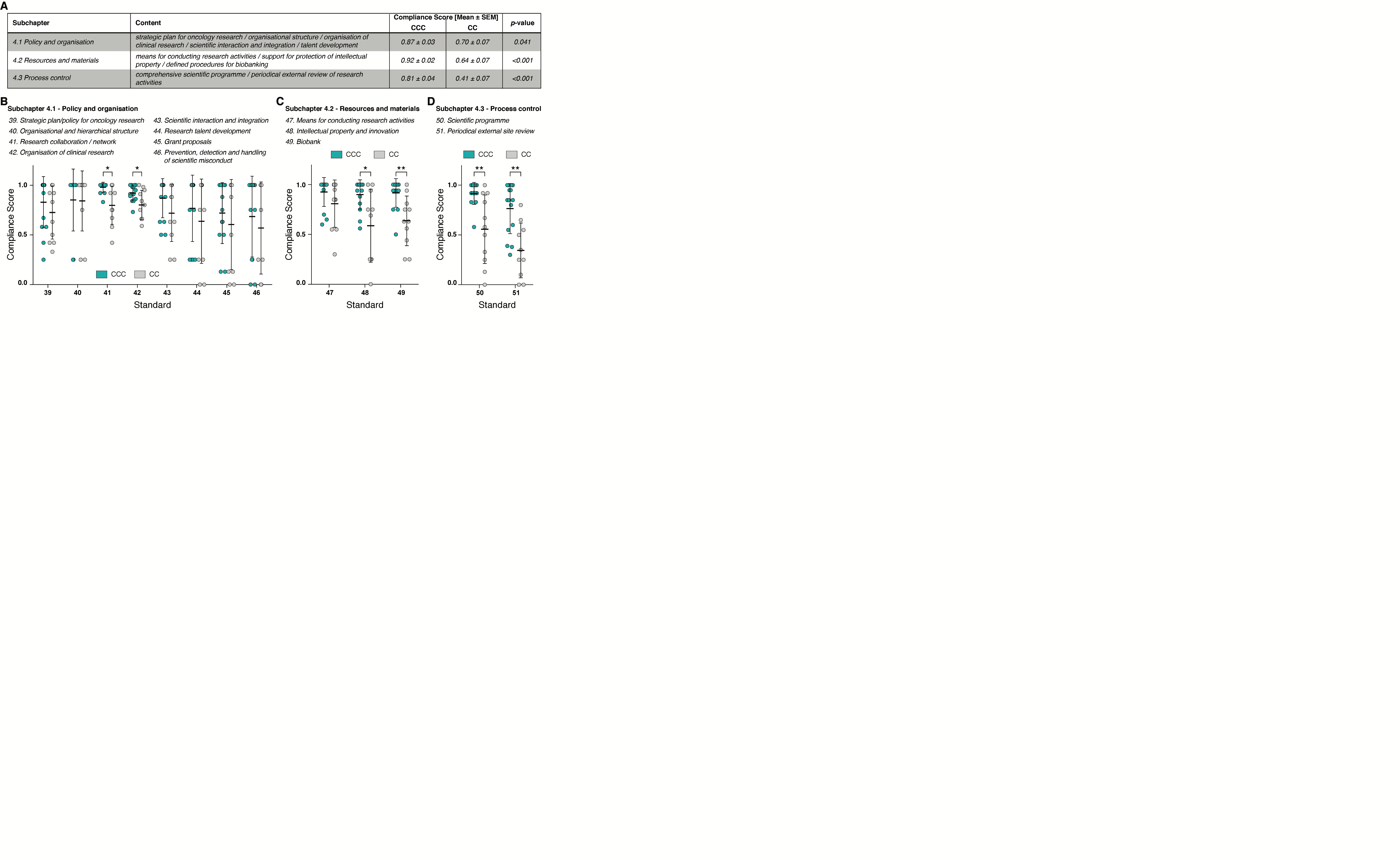

Supplement: Supplementary file 2 — Fig S2. Centre compliance to subcategories in chapters 4 – Research, Innovation and Development. [file MOL2-15-1277-s002.tiff]

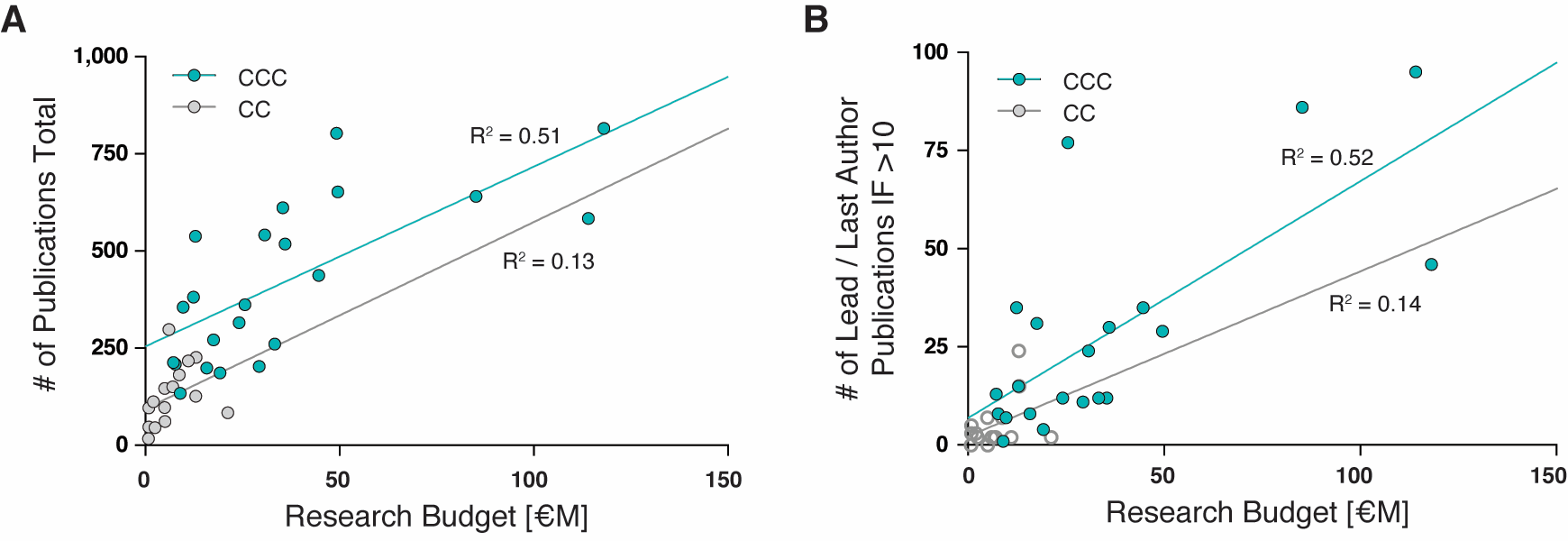

Supplement: Supplementary file 3 — Fig S3. Correlation of publication output with research budget. [file MOL2-15-1277-s003.tiff]
