# Supplementary material for: Analysing the attributes of Comprehensive Cancer Centres and Cancer Centres across Europe to identify key hallmarks
Source: Mol Oncol. 2021 Mar 30;15(5):1277–88. doi: 10.1002/1878-0261.12950 (PMC8096787; doi:10.1002/1878-0261.12950)
Supplement: Supplementary file 4 — Appendix S1. Background to the Accreditation and Designation (A&D) Programme of the Organisation of European Cancer Institutes. Table S1. List of accredited OECI Centres. Table S2. List of accredited OECI Centres. [file MOL2-15-1277-s004.docx]

Background to the Accreditation and Designation (A&D) Programme of the Organisation of European Cancer Institutes

OECI is a non-governmental, non-profit organization established in 1979 to promote collaboration among European cancer Centres and research institutes. There are 102 OECI member centres, 40 of which have completed at least one accreditation cycle. These 40 centres are active in 15 out of 27 Member States of the EU plus Norway, Turkey and the UK. They produce more than 12,400 peer reviewed publications on cancer research annually, have total annual research budgets of well over €1 billion, and have treated more than 1 million new patients since their accreditations. Though these accredited centres cover a small minority of cancer patients diagnosed each year in the EU, their impact on the quality of cancer care, through often being reference centres in their nations, is highly significant.

In 2002 the OECI initiated its Accreditation and Designation (A&D) Programme with the goal to i) provide cancer patients equal access to high-quality cancer care, ii) help European centres implement a quality control system, and iii) enhance translational cancer research. After a pilot period the A&D programme was formally launched in 2008. 52 centres are currently within the programme. The programme recognises two designation types, mainly clinically oriented OECI Cancer Centres (CCs) and OECI Comprehensive Cancer Centres (CCCs) (see Tables S1 and S2). Since 2002 the accreditation manual consisting of two separate questionnaires addressing quantitative and qualitative characteristics, has been revised twice, with Manual v2.0 being introduced in 2015^29^, and v3.0 being introduced in 2020.

During the A&D process each centre is reviewed individually based on the self-assessment provided through the questionnaires, followed by a site visit by a multidisciplinary OECI audit team, selected from other OECI centres. Centre status is designated based on certain thresholds and overall consistent performance (Table S2). But designation is only one endpoint of the process; the more dynamic and impactful goal is the requirement to agree and implement an Improvement Action Plan, mutually agreed between the centre and OECI, to address the opportunities for improvement as identified in the final report.

The OECI standards themselves have been accredited by the International Society for Quality in Health Care, guaranteeing a rigorous process of objectivity and evaluation. As part of the latest revision of the quality standards, 100 European core quality standards for cancer care and research centres have recently been published^28^. Directed towards the goal of improving the quality of patient diagnosis, treatment and care, the OECI Manual 2.0^29^ is divided into six chapters:

*Chapter 1: Leadership and management of the cancer centre*

*Chapter 2: Prevention and early diagnosis*

*Chapter 3: Cancer treatment and care*

*Chapter 4: Research, innovation and development*

*Chapter 5: Teaching and continuing education*

*Chapter 6: Patient centeredness*

The quality standards which form the basis of the programme fall into the three broad domains of care, research and education:

1. Multidisciplinarity, pathway-based and patient-centered care

High quality care is characterised by the integration of all necessary disciplines to cover the whole patient pathway within multidisciplinary teams (MDTs) or integrated practice units. Standards which test the quality of multidisciplinary care are within chapters 1, 2, 3 and 6 of the OECI Manual. Recommendations on patient treatment plans should be made in every case by MDTs, which at least consist of medical and radiation oncologists, surgical oncologists, radiologists, pathologists, nurses, and may include other supportive disciplines (such as psychologists, dieticians, physiotherapists, as appropriate). It is useful for triallists or principal investigators to attend and advise on recruitment of patients to relevant clinical trials, and increasingly the input of oncogenomics expertise is required. Shared decision-making should be the culture and practice of the centre.

1. Comprehensive research programme

This domain is addressed in chapter 4 of the OECI Manual, where the standards evaluate the range of translational research. Many centres integrate hospital oncology services with university-based research, providing a synergy between science and the clinic. OECI standards test the centres’ strategies to foster innovation, bridge translational gaps and ultimately support commercialisation of new drugs, tests or technologies. They examine the quality of processes in clinical research from early to late clinical development, and whether a significant percentage of patients are offered trials and benefit from novel treatment approaches. The standards also test the quality of enabling infrastructures such as clinical cancer registries, extensive biobanks and information management systems including electronic health records.

1. Comprehensive education programme

The educational aspects within a centre are tested in chapter 5 of the OECI Manual. Standards cover educational programmes for all professions involved in cancer care and research at all career levels, dissemination of knowledge and innovation within the wider healthcare system, and education of patients and their carers about the nature of cancer, treatment options, and information on survivorship and self-management.

**Methods**

The Qualitative Data we analysed relate to the auditors’ scoring of the degree of compliance with quality standards comprising 272 sub-questions within the six chapters of the manual. The full set of these subquestions can be referred to in the OECI Manual 2.0^29^. The starting point for the analysis was the entire data set for the 40 centres, subsequently selecting for chapters, sub-chapters, and individual standards where the scoring of the two cohorts (CCCs and CCs) showed statistically different results or wide ranges.

Quantitative data sets were selected (out of more than 800 possible metrics) based on their relevance to research, with the addition of some basic volume metrics.

We used the latest accreditation data set in every case, and we note that the base data of centres peer reviewed at different times can vary by as much as five years (Table S1). Data sets have been reviewed across all 40 centres to assure completeness and reliability. Outliers that have been identified have been verified and, if needed, manually curated by contacting the individual centres to validate the initially recorded data. Data that could not be confirmed have been excluded from statistical analyses as indicated in the figure captions. In view of the large number of items we only report on significant findings in this paper.

#### Statistical analysis of qualitative and quantitative data

Individual (sub)-standards in the qualitative questionnaires are scored by auditors as “yes”, “mostly”, “partially” and “no”. Data are recorded as percentages of substandards (individual questions) answered with the particular score. In order to compare across centres we calculated a compliance score using the following formula:


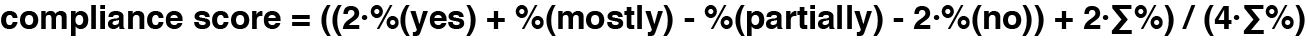


This is a standard methodology where the goal is to arrive at a normalized score from 0 to 1, 1 being 100% of available answers being “yes” and 0 being 100% of available answers being “no”. Normalisation to the sum of percentages answered no, partially, mostly or yes was necessary due to missing values or not-applicable standards for certain centres. Data points with more than 33% of missing values have been excluded from the analysis for the particular (sub-)chapter or standard.

Resulting compliance scores for chapters, subchapters and standards have been compared across CCCs and CCs using Welch’s t-test to account for heteroscedasticity of the data and followed by Bonferroni correction for multiple testing as indicated. Unbiased analysis was performed on chapters first, and if statistically significant differences between the two cohorts was found, analysis was then performed on sub-chapters, and thence to individual standards so as to identify the root sources of the differences between the centre types. At sub-chapter level, data were limited to the 19 centres assessed according to Manual 2.0 (Manual 1.0 contained a different selection of Standards).

Quantitative data on 22 CCCs and 18 CCs have been compared using Mann-Whitney test to account for non-normal distribution of the data. Financial data in Euros were corrected for purchasing power parity (PPP) with EU28 = 1^31^.

**Table S1: List of accredited OECI Centres.** Centres are listed in alphabetical order by country. Order does not indicate Centre numbers shown in Figure 2B. Asterisk indicates ongoing accreditation process, indicated designation is thus preliminary.

| **Centre Name** | **Country** | **City** | **Designation Year** | | **Designation** | |
| --- | --- | --- | --- | --- | --- | --- |
| AZ Groeninge | Belgium | Kortrijk |  | 2018 |  | CC |
| Brussel Cancer Centre | Belgium | Brussels |  | 2015 |  | CC |
| Institut Jules Bordet | Belgium | Brussels |  | 2018 |  | CCC |
| Masaryk Memorial Cancer Institute | Czech Republic | Brno |  | 2017 |  | CC |
| Vejle Cancer Centre – The Patients’ Cancer Hospital part of Lillebaelt Hospital | Denmark | Vejle |  | 2016 |  | CC |
| Foundation Tartu University Hospital | Estonia | Tartu |  | 2015 |  | CC |
| Helsinki University Hospital (HUS) | Finland | Helsinki |  | 2014 |  | CCC |
| TAYS Cancer Centre | Finland | Tampere |  | 2019 |  | CC |
| TYKS Cancer Centre | Finland | Turku |  | 2019 |  | CC |
| Centre Léon Bérard | France | Lyon |  | 2017 |  | CCC |
| Insitut Gustave Roussy | France | Villejuif |  | 2013 |  | CCC |
| Institut Curie | France | Paris |  | 2018 |  | CCC |
| Institut Paoli Calmettes | France | Marseille |  | 2019 |  | CCC |
| National Institute of Oncology | Hungary | Budapest |  | 2018 |  | CCC |
| Trinity St James's Cancer Institute | Ireland | Dublin |  | 2019 |  | CC |
| Azienda Unità Sanitaria Locale di Reggio Emilia - IRCCS Istituto in Tecnologie Avanzate e Modelli Assistenziali in Oncologia | Italy | Reggio Emilia |  | 2014 |  | CC |
| CRO Centro di Riferimento Oncologico Istituto Nazionale Tumori | Italy | Aviano |  | 2014 |  | CCC |
| Fondazione IRCCS Istituto Nazionale dei Tumori di Milano | Italy | Milan |  | 2015 |  | CCC |
| IEO European Institute of Oncology | Italy | Milan |  | 2020 |  | CCC |
| IRCCS Ospedale Policlinico San Martino | Italy | Genova |  | 2015 |  | CCC |
| IRCCS CROB Centro di Riferimento Oncologico della Basilicata | Italy | Rionero in Vulture |  | 2015 |  | CC |
| IRCCS Instituto Clinico Humanitas Cancer Centre | Italy | Milan |  | 2017 |  | CCC |
| Istituto Nazionale Tumori – IRCCS "Fondazione G. Pascale" | Italy | Naples |  | 2015 |  | CC |
| Istituto Nazionale Tumori Regina Elena | Italy | Rome |  | 2015 |  | CCC |
| Istituto Oncologico Veneto IRCCS-IOV | Italy | Padova |  | 2015 |  | CCC |
| Istituto Tumori Giovanni Paolo II, IRCCS | Italy | Bari |  | 2015 |  | CC |
| National Cancer Institute Vilnius | Lithuania | Vilnius |  | 2018 |  | CC |
| Maastricht University Medical Centre (MUMC) | Netherlands | Maastricht |  | 2020* |  | CCC |
| Netherlands Cancer Institute (NKI) | Netherlands | Amsterdam |  | 2017 |  | CCC |
| Oslo University Hospital (OUH) | Norway | Oslo |  | 2017 |  | CCC |
| Instituto Português de Oncologia de Coimbra Francisco Gentil, EPE (IPO-Coimbra) | Portugal | Coimbra |  | 2017 |  | CC |
| Instituto Português de Oncologia de Lisboa Francisco Gentil, EPE (IPO-Lisboa) | Portugal | Lisbon |  | 2017 |  | CC |
| Instituto Português Oncologia do Porto Francisco Gentil, EPE (IPO-Porto) | Portugal | Porto |  | 2017 |  | CCC |
| Oncology Institute "Prof. Dr. Ion Chiricuta" | Romania | Cluj |  | 2017 |  | CC |
| Fundacion Instituto Valenciano de Oncologia | Spain | Valencia |  | 2018 |  | CC |
| Karolinska Institutet | Sweden | Stockholm |  | 2020 |  | CCC |
| Anadolu Medical Centre | Turkey | Kocaeli |  | 2018 |  | CC |
| Cambridge Cancer Centre | UK | Cambridge |  | 2020 |  | CCC |
| King's Health Partners Integrated Cancer Centre | UK | London |  | 2015 |  | CCC |
| The Christie NHS Foundation Trust | UK | Manchester |  | 2018 |  | CCC |

**Table S2: OECI Designation criteria for CCs and CCCs in Manual 2.**

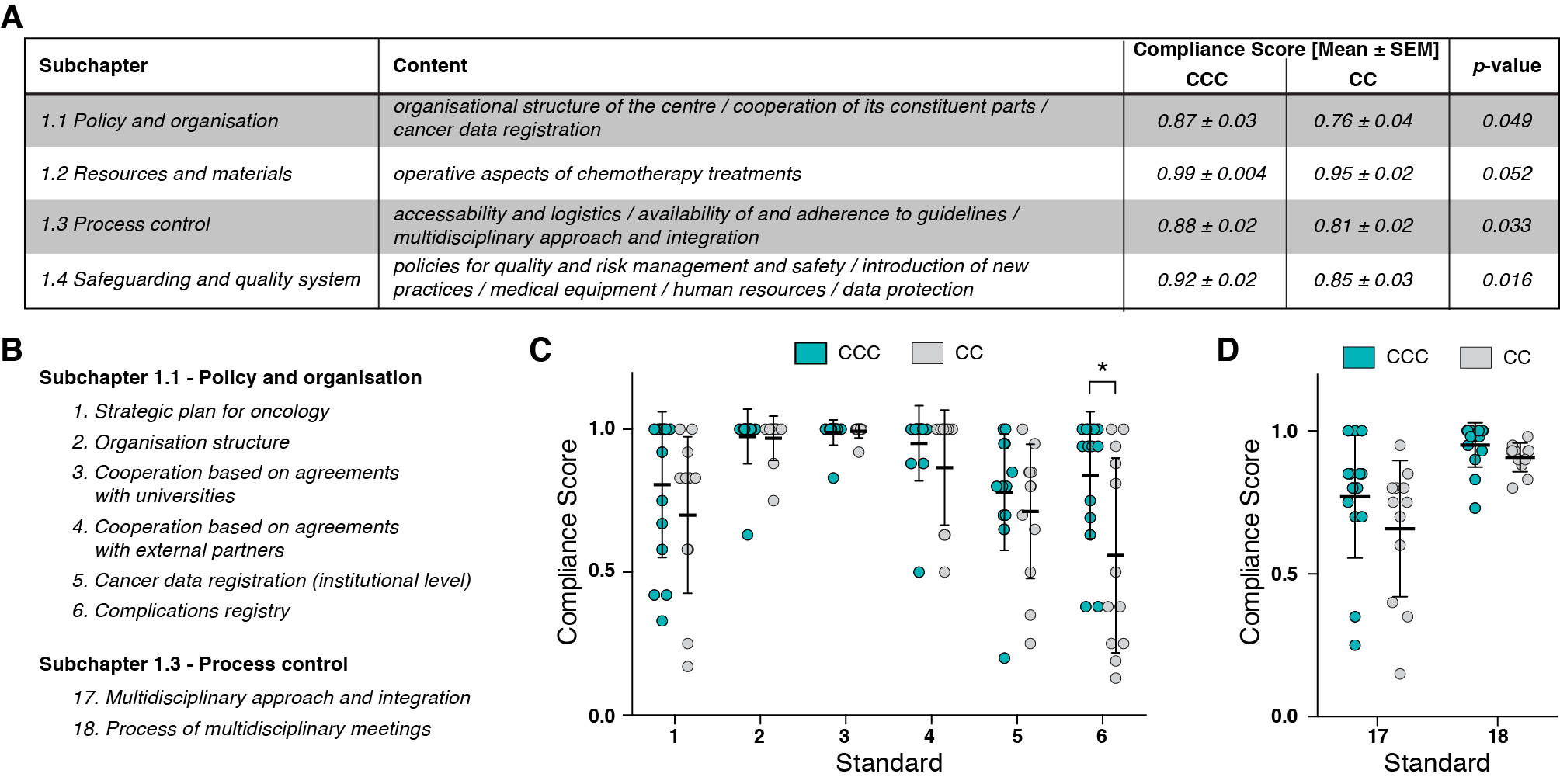


**Figure S1: Centre compliance to subcategories in chapters 1 – Leadership and Management.** Compliance was compared between the two designation types across subchapters 1.1-1.4 (A). Compliance scores for individual standards of subchapters 1.1 and 1.3 (B) are shown in (C) and (D). (CCC n=15, CC n=12, data limited to centres accredited under A&D Manual v2.0). Middle horizontal lines represent the mean, and error bars represent the standard deviation, ** P < 0.05* (Welch’s t-test)*.*

----------------------------------------------------------------------------------------------------------------------------------------------


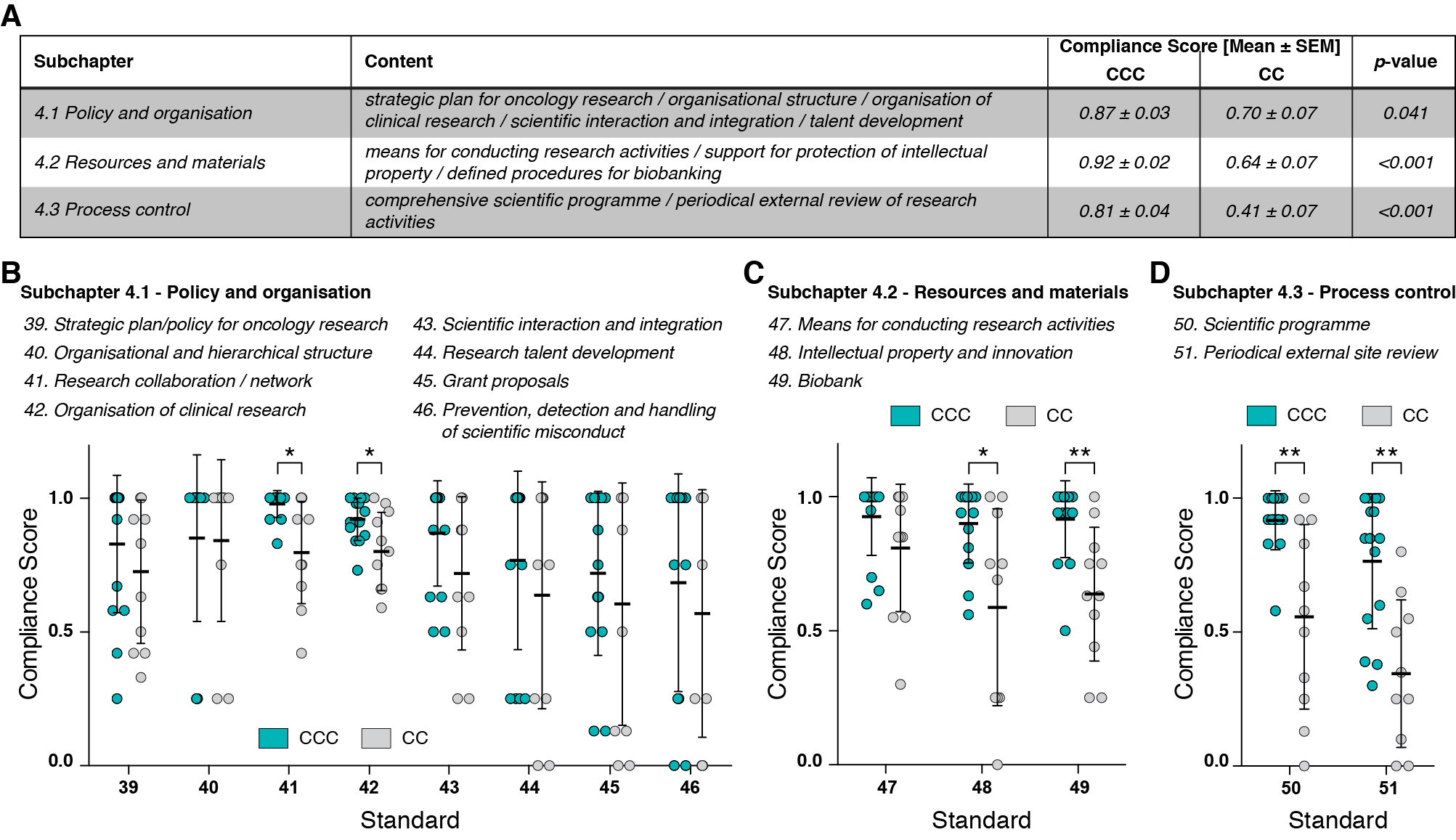


**Figure S2: Centre compliance to subcategories in chapters 4 – Research, Innovation and Development.** Compliance was compared between the two designation types across subchapters 4.1-4.3 (**A**). Compliance scores for individual standards are shown in (**B**), (**C**) and (**D**). (CCC n=15, CC n=11, data limited to centres accredited under A&D Manual v2.0). Middle horizontal lines represent the mean, and error bars represent the standard deviation, ** P < 0.05, ** P < 0.01* (Welch’s t-test)*.*

**
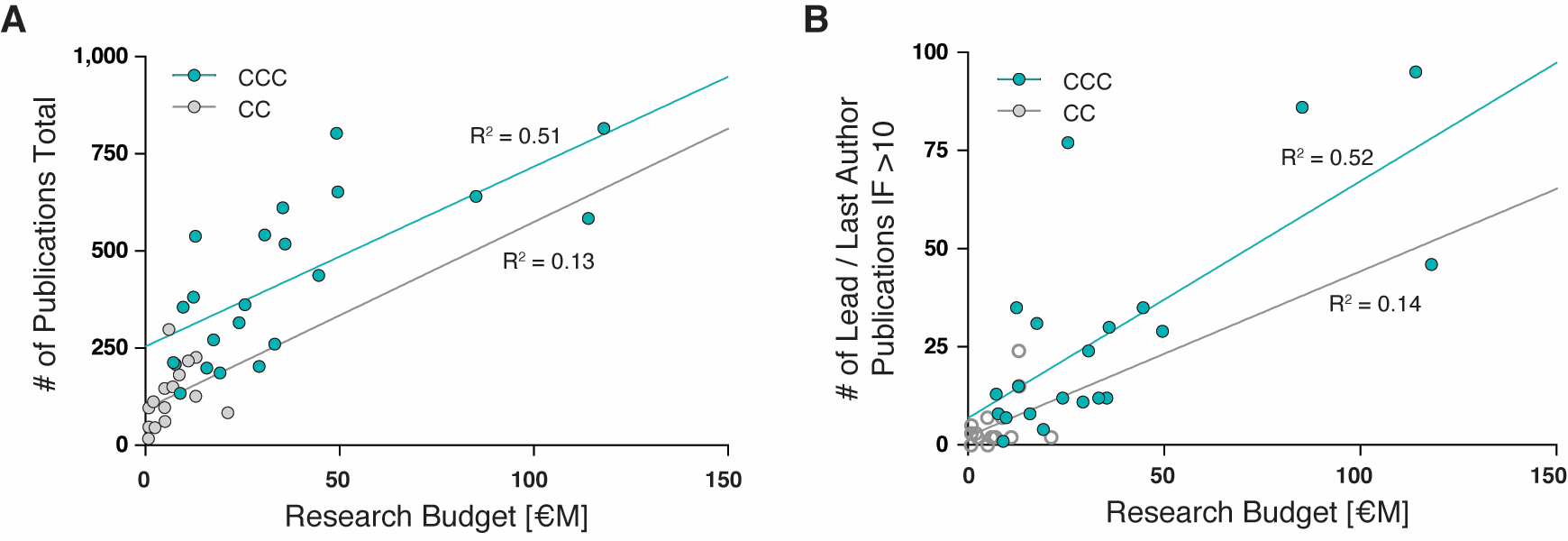
**

**Figure S3: Correlation of publication output with research budget.** Total publication output (**A**) and number of high impact publications with first or last author from the accredited centre (**B**) was correlated with the centre’s research budget normalised by PPP. Goodness-of-fit of linear regression is indicated by R square values next to the fitted lines.
